# *HNF4A* mutation: switch from hyperinsulinaemic hypoglycaemia to maturity-onset diabetes of the young, and incretin response

**DOI:** 10.1111/dme.12369

**Published:** 2014-02-16

**Authors:** V B Arya, S Rahman, S Senniappan, S E Flanagan, S Ellard, K Hussain

**Affiliations:** 1 Department of Paediatric Endocrinology, Great Ormond Street Hospital for Children NHSTrust, London; 2 The Institute of Child Health, University College LondonExeter, UK; 3 Institute of Biomedical and Clinical Science, University of Exeter Medical SchoolExeter, UK

## Abstract

**Background:**

Hepatocyte nuclear factor 4α (HNF4A) is a member of the nuclear receptor family of ligand-activated transcription factors. *HNF4A* mutations cause hyperinsulinaemic hypoglycaemia in early life and maturity-onset diabetes of the young. Regular screening of *HNF4A* mutation carriers using the oral glucose tolerance test has been recommended to diagnose diabetes mellitus at an early stage. Glucagon-like peptide-1 and glucose-dependent insulinotropic polypeptide are incretin hormones, responsible for up to 70% of the secreted insulin after a meal in healthy individuals. We describe, for the first time, gradual alteration of glucose homeostasis in a patient with *HNF4A* mutation after resolution of hyperinsulinaemic hypoglycaemia, on serial oral glucose tolerance testing. We also measured the incretin response to a mixed meal in our patient.

**Case report:**

Our patient was born with macrosomia and developed hyperinsulinaemic hypoglycaemia in the neonatal period. Molecular genetic analysis confirmed *HNF4A* mutation (p.M116I, c.317G>A) as an underlying cause of hyperinsulinaemic hypoglycaemia. Serial oral glucose tolerance testing, after the resolution of hyperinsulinaemic hypoglycaemia, confirmed the diagnosis of maturity-onset diabetes of the young at the age of 10 years. Interestingly, the intravenous glucose tolerance test revealed normal glucose disappearance rate and first-phase insulin secretion. Incretin hormones showed a suboptimal rise in response to the mixed meal, potentially explaining the discrepancy between the oral glucose tolerance test and the intravenous glucose tolerance test.

**Conclusions:**

Maturity-onset diabetes of the young can develop as early as the first decade of life in persons with an *HNF4A* mutation. Impaired incretin response might be contributory in the early stages of *HNF4A* maturity-onset diabetes of the young.

## Introduction

Hepatocyte nuclear factor 4α (HNF4A) is a member of the steroid/thyroid hormone receptor superfamily and is most highly expressed in the liver, kidney, pancreatic islets and intestine [1,2]. HNF4A is a major activator of hepatocyte nuclear factor 1α (HNF1A), which in turn activates the expression of a large number of liver-specific genes, including those involved in glucose, cholesterol and fatty acid metabolism [3]. Specifically in the pancreatic islets, HNF4A has been shown to control the expression of genes involved in glucose-stimulated insulin secretion [4,5]. *HNF4A* mutations cause hyperinsulinaemic hypoglycaemia and maturity-onset diabetes of the young (MODY) [6–8]. Annual screening of *HNF4A-*mutation carriers with oral glucose tolerance testing was recommended to diagnose diabetes mellitus at an early stage [7].

Glucagon-like peptide-1 (GLP-1) and glucose-dependent insulinotropic polypeptide are incretin hormones, which augment the magnitude of meal-stimulated insulin secretion from islet β-cells in a glucose-dependent manner [9].

We describe, for the first time, the gradual alteration in glucose homeostasis and development of MODY in the *HNF4A-*mutation carrier after resolution of hyperinsulinaemic hypoglycaemia, on prospective follow-up with an annual oral glucose tolerance test. We also report the GLP-1 and glucose-dependent insulinotropic polypeptide response to the mixed-meal test at the onset of diabetes mellitus.

**What's new?**

- This study reports the first patient with an *HNF4A* mutation to have been serially monitored from the diagnosis of hyperinsulinaemic hypoglycaemia to maturity-onset diabetes of the young.
- We describe, for the first time, incretin response in a patient with *HNF4A* maturity-onset diabetes of the young, which has the potential to influence the management of these patients.

## Research design and methods

### *KCNJ11, ABCC8* and *HNF4A* sequencing

Genomic DNA was extracted from peripheral leukocytes using standard procedures, and the coding exons and intron/exon boundaries of the *ABCC8*,*KCNJ11* and *HNF4A* genes were amplified by polymerase chain reaction (PCR). *HNF4A* analysis included the coding exons 1d–10 and the P2 pancreatic promoter. PCR products were sequenced using standard methods on an ABI 3730 sequencer (Applied Biosystems, Warrington, UK) and were compared with the published sequence NM_000457.3 (exons 2–10) and AY680697 (exon 1d only) using Mutation Surveyor v3.2 (SoftGenetics, State College, PA, USA) [10].

### Oral glucose tolerance test

An oral glucose tolerance test (glucose load 1.75 g/kg) was performed in accordance with standard recommendations and the results were interpreted according to published World Health Organization (WHO) diagnostic criteria [11,12].

### Intravenous glucose tolerance test

After an overnight fast of 12 h, 0.5 g/kg of glucose as a 20% solution was infused within 2 min through an intravenous catheter in the antecubital region. Blood samples were obtained at –10, –5, 0, 1, 3, 5, 10, 15, 20, 30, 45 and 60 min through a second intravenous catheter for estimation of blood glucose and serum insulin. The glucose disappearance rate *K* (%/min) was calculated according to ^0.693 × 100^/_*t*1/2_ (in min), where *t*_1/2_ was the time span (min) required for glucose to fall by 50% from its level at 10 min [12]. The first-phase insulin release was calculated by the area under the insulin stimulation curve between 0 and 10 min [12].

### Mixed-meal tolerance test

The mixed-meal tolerance test was performed after an overnight fast of 12 h. A standardized breakfast was ingested over 10 min, calculated by a dietician based on the total caloric needs of the patient (25–30% of their daily caloric intake; 50% of the calories as carbohydrates, 33% lipids and 17% proteins). Blood samples were collected at 0, 15, 30, 45 and 60 min for estimation of blood glucose, serum insulin, total glucose-dependent insulinotropic polypeptide and active GLP-1. The area under curve was estimated by the linear trapezoidal method from the concentration time curve.

### Measurement of GLP1 and glucose-dependent insulinotropic polypeptide

Blood was collected in chilled EDTA-treated tubes containing DPP-4 inhibitor (10 μl/ml blood: Millipore, Watford, UK) and aprotonin (trasylol 5000 KIU/ml blood: Bayer, Newbury, UK). Blood was processed according to the manufacturers’ instructions for the measurement of hormones using commercially available assays. Total glucose-dependent insulinotropic polypeptide and active GLP-1 were determined using commercially available human ELISA kits according to the manufacturer's guidelines (Millipore). All samples were performed in duplicate.

## Case report

We describe a male Caucasian patient, who was born with macrosomia [birthweight 4060 g at 39 weeks of gestation; +1.44 standard deviation score (SDS)] and who developed neonatal hypoglycaemia requiring a high glucose infusion rate (18.5 mg kg^–1^ min^–1^) to maintain normoglycaemia. There was no family history of diabetes mellitus. Investigations revealed inappropriately elevated serum insulin and inappropriately low β-hydroxybutyrate and non-esterified fatty acids during an episode of hypoglycaemia on three occasions, confirming hyperinsulinaemic hypoglycaemia. Serum cortisol, lactate, ammonia, plasma aminoacids, carnitine profile and urine organic acids were normal. Molecular genetic testing for mutation in *ABCC8/KCNJ11* was negative. Hyperinsulinaemic hypoglycaemia was successfully managed with diazoxide. A 24-h blood glucose profile and controlled fasting studies were carried out at regular intervals until the age of 18 months, at which age his hyperinsulinaemic hypoglycaemia resolved.

At the age of 5 years, after *HNF4A* mutations were first reported as a cause of macrosomia and neonatal hyperinsulinaemic hypoglycaemia, he underwent molecular genetic testing and was identified to have a *de novo* heterozygous *HNF4A* mutation (p.M116I, c.317G>A) [13].

### Oral glucose tolerance test

Annual monitoring with an oral glucose tolerance test revealed impaired glucose tolerance and development of diabetes mellitus according to WHO diagnostic criteria (Table 1) [11]. At the diagnosis of diabetes mellitus, he was prepubertal with a bilateral testicular volume of 2 ml. His HbA_1c_ was 40 mmol/mmol (5.8%) and autoantibodies (GAD 65 and islet-cell) were negative. His serial BMI and BMI SDS were normal (Table 1).

**Table 1:** Oral glucose tolerance test

Age BMI, kg/m^2^ (SDS)	7 years 15.25 (–0.22)		8 years 15.8 (+0.05)		9 years 17.6 (+0.75)		10 years 19.9 (+1.17)		
Time	Blood glucose (mmol/l)	Serum insulin (mU/l)	Blood glucose (mmol/l)	Serum insulin (mU/l)	Blood glucose (mmol/l)	Serum insulin (mU/l)	Blood glucose (mmol/l)	Serum insulin (mU/l)	C-peptide (pmol/l)
–30 min	4.0	< 2.0	4.0	< 2.0	4.3	< 2.0			
0 min	3.6	< 2.0	3.9	< 2.0	4.2	< 2.0	4.8	5.6	440
30 min	8.8	39.8	8.6	39.6	6.9	23	9.2	47.1	1903
60 min	7.7	39.1	8.5	26.4	9.5	30	11.7	66.7	2522
90 min	6.7	22.5	6.8	17.0	8.5	21	11.5	44.1	2214
120 min	6.8	23.6	8.3	28.9	9.3	26	12.3	35.9	1956
150 min	3.4	6.6	6.2	13.8			13.3	36.3	1926
180 min	3.1	< 2.0	3.2	< 2.0			8.4	17.0	1202

### Intravenous glucose tolerance test

**Figure 1:**
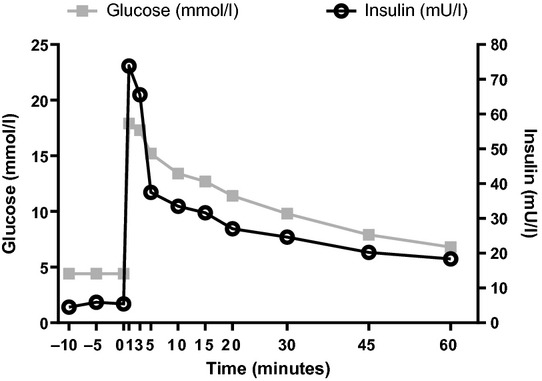
Intravenous glucose tolerance test.

An intravenous glucose tolerance test (Fig. 1) revealed normal glucose disappearance rate (1.38% per min; normal response > 1.2% per min) and first-phase insulin secretion (2.7 nmol l^−1^ min^−1^ normal 1.4–5.3 nmol l^–1^ min^–1^), which were not indicative of insulin secretion defect [12].

### Mixed-meal tolerance test

**Figure 2:**
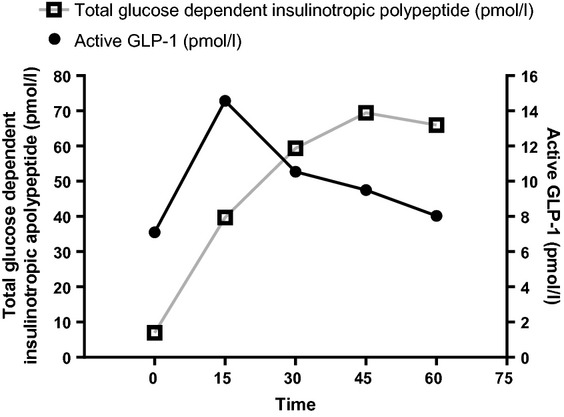
Total glucose-dependent insulinotropic polypeptide and active glucagon-like peptide 1 (GLP-1) response to the mixed-meal tolerance test.

To understand the reasons for the discrepancy between diabetic oral glucose tolerance test response and normal intravenous glucose tolerance test response, we measured the incretin hormones in response to the mixed-meal tolerance test (Fig. 2). The 30-min areas under the curve for active GLP-1 and total glucose-dependent insulinotropic polypeptide were 350.9 and 740.2 pmol/l, respectively, which are lower than the sparse data on these hormones in the literature in this age group.

## Discussion

MODY is a genetically heterogeneous monogenic form of diabetes mellitus, characterized by early onset, usually before 25 years of age and often in adolescence or childhood, and by autosomal dominant inheritance [14]. Heterozygous mutations in the genes encoding the glycolytic enzyme glucokinase and the transcription factors, HNF-1α, HNF-4α and HNF1β have been shown to cause MODY. Clinical studies have shown normal insulin sensitivity, but reduced glucose-stimulated insulin secretion in subjects with pre-diabetes with *HNF-4A* MODY, suggesting that pancreatic β-cell dysfunction rather than insulin resistance is the primary defect in this disorder [15].

Recently, *HNF4A* mutations have been shown to cause macrosomia and hyperinsulinaemic hypoglycaemia (transient and persistent) in early life [7,8]. Hence, in *HNF4A* mutation carriers, there would need to be a switch from increased insulin secretion *in utero* and neonatal life to decreased glucose-stimulated insulin secretion in later life. The molecular basis for the differential aspects of HNF-4α deficiency on β-cell function and insulin secretion in neonates and later in life and the exact timing of the switch from hyperinsulinaemia to hypoinsulinaemia are unknown.

We describe the first patient with an *HNF4A* mutation to have been serially monitored with oral glucose tolerance testing through to MODY from hyperinsulinaemic hypoglycaemia. The patient was macrosomic at birth and exhibited hyperinsulinaemic hypoglycaemia in the neonatal period and infancy. Annual oral glucose tolerance test monitoring highlighted gradually worsening glucose tolerance (impaired oral glucose tolerance test). At age 10 years, he fulfilled the WHO oral glucose tolerance test criteria for diabetes mellitus [11].

Surprisingly, intravenous glucose tolerance testing demonstrated a normal glucose disappearance rate (*K*) and first-phase insulin secretion, contradictory to what one would expect in MODY (*K* 1.38% per min; normal response > 1.2%, first-phase insulin secretion 2.7 nmol l^–1^ min^–1^; normal 1.4–5.3 nmol l^–1^ min^–1^). Similar to our results, when assessed by intravenous glucose tolerance test, Herman *et al*. [15] did not find any significant difference in insulin secretion in *HNF4A* mutation carriers without diabetes as compared with control subjects either. As our patient has been diagnosed at a very early stage of MODY, it is possible that his insulin secretion as assessed by intravenous glucose tolerance test is still preserved.

As our patient had a diabetic response to the oral glucose tolerance test and a relatively normal response to the intravenous glucose tolerance test, we hypothesized a suboptimal glucose-dependent insulinotropic polypeptide and GLP-1 rise in response to oral glucose/meal in *HNF4A* mutation carriers. Glucose-dependent insulinotropic polypeptide and GLP-1 are peptide hormones, responsible for up to 70% of the secreted insulin after a meal in healthy individuals [16].

In response to a mixed-meal tolerance test, the 30-min areas under the curve for GLP-1 and glucose-dependent insulinotropic polypeptide were 350.9 and 740.2 pmol/l, respectively. Rask *et al*. reported a 30-min area under the curve for GLP-1 and glucose-dependent insulinotropic polypeptide after oral glucose as 460 ± 60 and 1491 ± 136 pmol/l, respectively, in subjects with impaired glucose tolerance [17]. Huml *et al*. measured GLP-1 and glucose-dependent insulinotropic polypeptide in healthy children, but at a single time point of 90 min after the standardized breakfast [18]. Unfortunately, there are no normative data on GLP-1 and glucose-dependent insulinotropic polypeptide in healthy control children. Comparing the results, it does seem that our patient mounted a lower GLP-1 and glucose-dependent insulinotropic polypeptide response to the mixed-meal tolerance test, potentially explaining the discrepancy for responses to the oral glucose tolerance test and intravenous glucose tolerance test. However, there are limitations to comparing our results with adult subjects with impaired glucose tolerance from another cause.

From our data, it is plausible that incretin response might be impaired and contributory to glucose intolerance in the preclinical stages in some patients with *HNF4A* MODY. Once glucose intolerance sets in, chronic exposure to supraphysiologic concentrations of glucose over several months can lead to gradual loss of insulin gene expression [19,20].

In conclusion, we report the first patient with *HNF4A* hyperinsulinaemic hypoglycaemia to have switched to maturity-onset diabetes of the young on serial oral glucose tolerance testing. Impaired glucose tolerance can develop within the first decade of life in these patients. Our findings suggest a possibility of impaired incretin response and this is an exciting area to be explored in *HNF4A* maturity-onset diabetes of the young. More *HNF4A*-positive subjects with pre-diabetes and diabetes need to be studied to understand the impact of the *HNF4A* mutation on incretin effect.

## Funding sources

The genetic testing was funded by a Medical Council Grant award.

## Competing interests

None declared.

## References

[1] Xanthopoulos KG, Prezioso VR, Chen WS, Sladek FM, Cortese R, Darnell JE (1991). The different tissue transcription patterns of genes for HNF-1, C/EBP, HNF-3, and HNF-4, protein factors that govern liver-specific transcription. Proc Natl Acad Sci U S A.

[2] Sladek FM, Zhong WM, Lai E, Darnell JE (1990). Liver-enriched transcription factor HNF-4 is a novel member of the steroid hormone receptor superfamily. Genes Dev.

[3] Kuo CJ, Conley PB, Chen L, Sladek FM, Darnell JE, Crabtree GR (1992). A transcriptional hierarchy involved in mammalian cell-type specification. Nature.

[4] Odom DT, Zizlsperger N, Gordon DB, Bell GW, Rinaldi NJ, Murray HL (2004). Control of pancreas and liver gene expression by HNF transcription factors. Science.

[5] Wang H, Maechler P, Antinozzi PA, Hagenfeldt KA, Wollheim CB (2000). Hepatocyte nuclear factor 4α regulates the expression of pancreatic β-cell genes implicated in glucose metabolism and nutrient-induced insulin secretion. J Biol Chem.

[6] Yamagata K, Furuta H, Oda N, Kaisaki PJ, Menzel S, Cox NJ (1996). Mutations in the hepatocyte nuclear factor-4α gene in maturity-onset diabetes of the young (MODY1). Nature.

[7] Pearson ER, Boj SF, Steele AM, Barrett T, Stals K, Shield JP (2007). Macrosomia and hyperinsulinaemic hypoglycaemia in patients with heterozygous mutations in the HNF4A gene. PLoS Med.

[8] Kapoor RR, Locke J, Colclough K, Wales J, Conn JJ, Hattersley AT (2008). Persistent hyperinsulinemic hypoglycemia and maturity-onset diabetes of the young due to heterozygous HNF4A mutations. Diabetes.

[9] Drucker DJ (2007). The role of gut hormones in glucose homeostasis. J Clin Invest.

[10] Colclough K, Bellanne-Chantelot C, Saint-Martin C, Flanagan SE, Ellard S (2013). Mutations in the genes encoding the transcription factors hepatocyte nuclear factor 1 alpha and 4 alpha in maturity-onset diabetes of the young and hyperinsulinemic hypoglycemia. Hum Mutat.

[11] Alberti KG, Zimmet PZ (1998). Definition, diagnosis and classification of diabetes mellitus and its complications. Part 1: diagnosis and classification of diabetes mellitus provisional report of a WHO consultation. Diabet Med.

[12] Heinze E, Holl RW, Ranke MB (2003). Test of β-cell function in childhood and adolescence. Diagnostics of Endocrine Function in Children and Adolescents.

[13] Flanagan SE, Kapoor RR, Mali G, Cody D, Murphy N, Schwahn B (2010). Diazoxide-responsive hyperinsulinemic hypoglycemia caused by HNF4A gene mutations. Eur J Endocrinol.

[14] Fajans SS (1989). Maturity-onset diabetes of the young (MODY). Diabetes Metab Rev.

[15] Herman WH, Fajans SS, Ortiz FJ, Smith MJ, Sturis J, Bell GI (1994). Abnormal insulin secretion, not insulin resistance, is the genetic or primary defect of MODY in the RW pedigree. Diabetes.

[16] Herzberg-Schafer S, Heni M, Stefan N, Haring HU, Fritsche A (2012). Impairment of GLP1-induced insulin secretion: role of genetic background, insulin resistance and hyperglycaemia. Diabetes Obes Metab.

[17] Rask E, Olsson T, Soderberg S, Holst JJ, Tura A, Pacini G (2004). Insulin secretion and incretin hormones after oral glucose in non-obese subjects with impaired glucose tolerance. Metab Clin Exp.

[18] Huml M, Kobr J, Siala K, Varvarovska J, Pomahacova R, Karlikova M (2011). Gut peptide hormones and pediatric type 1 diabetes mellitus. Physiol Res.

[19] Robertson RP, Zhang HJ, Pyzdrowski KL, Walseth TF (1992). Preservation of insulin mRNA levels and insulin secretion in HIT cells by avoidance of chronic exposure to high glucose concentrations. J Clin Invest.

[20] Olson LK, Redmon JB, Towle HC, Robertson RP (1993). Chronic exposure of HIT cells to high glucose concentrations paradoxically decreases insulin gene transcription and alters binding of insulin gene regulatory protein. J Clin Invest.

